# Risk Factors Associated With Intrauterine Growth Restriction: A Case-Control Study

**DOI:** 10.7759/cureus.40178

**Published:** 2023-06-09

**Authors:** Priyanka Dapkekar, Anuja Bhalerao, Anjali Kawathalkar, Nikita Vijay

**Affiliations:** 1 Department of Obstetrics and Gynaecology, NKP Salve Institute of Medical Sciences and Research Centre, Nagpur, IND

**Keywords:** perinatal or neonatal mortality, preeclampsia, anemia, foetal growth restriction, intrauterine growth restriction (iugr)

## Abstract

Background

Intrauterine growth restriction (IUGR) is a disorder in which the fetus fails to reach its genetic development potential and is considered to be present when the weight at birth is less than the 10th percentile; as a result, it is at risk of increased postnatal morbidity and mortality. Every year, approximately 24% of newborns worldwide are determined to have IUGR. The objective of the present study was to identify various sociodemographic, medical, and obstetric risk factors associated with IUGR.

Methodology

A case-control study was conducted from January 2020 to December 2022. Fifty-four cases and 54 controls were included in the study. Postnatal women with neonates having birth weight below the 10th percentile for gestational age (GA) were recruited as cases in the study. Control cases were postnatal women with neonatal birth weight appropriate for (GA). Detailed history with respect to socio-demographic, medical, and obstetric parameters was noted and compared.

Results

Among the sociodemographic factors, only socioeconomic status showed significant statistical differences with the age group of 21 to 25 years showing maximum (51.9%) IUGR cases. Among the maternal risk factors, anemia (29.6%) and hypertensive disorders of pregnancy (22.2%) were marked as significant risk factors for IUGR. There was no significant difference in the distribution of past medical and obstetric histories between the two research groups.

Conclusion

Due to the poor living conditions, low literacy rates, and general lack of knowledge, low socioeconomic level increases the risk of IUGR. This leads to nutritional deficiencies and insufficient growth environment which results in anemia and hypertensive disorders of pregnancy which are potent risk factors for IUGR. IUGR may be caused by maternal risk factors as well as past medical and obstetric conditions. However, for the risk factor of IUGR, the birth weight at the time of delivery could be taken into consideration as well.

## Introduction

Intrauterine growth restriction (IUGR) is a condition where the fetus fails to achieve its genetic growth potential and consequently is at risk of increased perinatal morbidity and mortality [1]. Fetal growth restriction (FGR) is considered to be present when weight at birth is less than the 10th percentile of the average for the gestational age. About 24% of newborns are found to have IUGR over the world and every year, 30 million infants are affected [2]. After prematurity, FGR is the second most prevalent factor causing perinatal morbidity and mortality [3]. Despite improvements in obstetric care, IUGR is still common in developing nations. The reasons for IUGR in these regions, however, differ from those in developed nations. The primary cause of IUGR in the majority of Western societies is placental insufficiency; however, in developing nations, malnutrition and malaria infections are more significant contributors [4]. Anemia is more prevalent in low-income nations with a higher proportion of low birth weight outcomes [5].

With a 10-15% incidence rate among pregnant women, IUGR is one of the significant public health problems [6]. At the initial antenatal visit, all pregnant women undergo a thorough medical history gathered because determining risk factors is easy and inexpensive. The majority of maternal risk factors are modifiable. IUGR has the potential to cause a wide range of complications throughout the antenatal, intrapartum, or postnatal periods. Hence, the present study has been designed to identify the risk factors for IUGR which could be addressed to reduce infant mortality and morbidity.

## Materials and methods

This case-control study was approved by Institutional Ethics Committee (IEC), NKP Salve Institute of Medical Sciences and Research Centre, Nagpur, with reference number 96/2021. The study was conducted at the Department of Obstetrics and Gynaecology attached to the Tertiary Healthcare Centre and Teaching Institute from January 2021 To December 2022. A total of 54 cases with IUGR (Group A) and 54 controls from the labor room or obstetric ward (Group B) were recruited in accordance with the study's selection criteria. 

The selection criteria consisted of cases that were postpartum mothers with neonatal birth weights that were less than the 10th percentile for gestational age (GA) [7]. Controls were postnatal women with neonatal birth weight appropriate for GA delivering consecutive to the assigned cases. Using Naegele's method, GA was determined for cases treated with in vitro fertilization by counting the days since oocyte retrieval or co-incubation and then adding 14 days [8]. The collection of data was performed by an interview-based technique and by checking the files of the patients for both cases as well as the control group at the time of discharge of the patient and/or baby. Birth weight was measured immediately after delivery, without clothes using a weighing scale (Essae Electronics). Detailed history such as maternal age, socioeconomic status, parity, booking status, number of antenatal visits, and significant medical/obstetric history was noted.

Statistical Package for Social Sciences (SPSS) version 24.0 (IBM Corp., Armonk, NY) for MS Windows was used for the statistical analysis. The data on continuous variables are provided as mean and standard deviation (SD), whereas the data on categorical variables are displayed as n (percent of cases). If more than 20% of the cells have an expected frequency of less than 5, the chi-square test or Fisher's exact probability test is used to compare the distribution of categorical variables between groups. Using an independent sample t-test, the means of normally distributed continuous variables are statistically compared between groups.

## Results

Overall 108 women were recruited and divided into two groups of 54 each. Group A consisted of cases (IUGR group) whereas Group B consisted of controls (from the labor room or obstetric ward). In Group A, 51.9% belonged to the age group between 21-25 years, 37.0% belonged to the age group between 26-30 years, and 11.1% were above 30 years of age. Whereas, in Group B, 40.7% were in the age group between 21-25 years, 48.2% were in the age group between 26-30 years, and 11.1% were above 30 years of age. The age distribution of the case and control study populations did not differ substantially (p value>0.05) between the two groups.

In Group A, 29.3% of the women belonged to the upper lower class, while in Group B, 53.7% of the women in the control group belonged to the lower middle class, indicating a significant difference in the distribution between the cases and controls (p-value 0.05). However, no statistically significant difference was indicated by the distribution based on parity.

In order to identify the risk factors for IUGR, the maternal variables were evaluated. In comparison to controls in Group B, the prevalence of anemia and hypertensive disorders of pregnancy is significantly greater (p-value 0.05) among the cases studied in Group A. As demonstrated in Table 1, no statistically significant differences were found for the following conditions: multifetal pregnancy, diabetes mellitus, hypothyroidism, antepartum hemorrhage, uterine fibroids, or hypothyroidism.

**Table 1:** Inter-group comparison of maternal risk factors

Maternal factors	Group A (cases n=54)		Group B (controls n=54)		p-value
	n	%	n	%	
Anemia	16	29.6	7	12.9	0.034
Hypertensive disorders of pregnancy	12	22.2	3	5.6	0.023
Diabetes mellitus	10	18.5	4	7.4	0.15
Hypothyroidism	10	18.5	4	7.4	0.15
Antepartum hemorrhage	2	3.7	1	1.9	0.999
Fibroid uterus	1	1.9	0	0.0	0.999
Multifetal pregnancy	1	1.9	0	0.0	0.999

The distribution of maternal past medical and obstetric history included the history of perinatal/neonatal mortality, assisted reproductive technique, COVID-19, fever, sickle cell trait, abortion, and IUGR, demonstrating no significant difference between the two groups as shown in Table 2.

**Table 2:** Inter-group comparison of maternal past medical and obstetric history factors IUGR = intrauterine growth restriction

Maternal past medical and obstetric history factors	Group A (cases n=54)		Group B (controls n=54)		p-value
	n	%	n	%	
Perinatal/neonatal mortality	3	5.6	4	7.4	0.999
Assisted reproductive technique	4	7.4	0	0.0	0.118
COVID-19	1	1.9	0	0.0	0.999
Fever	6	11.1	3	5.6	0.489
Sickle cell trait	3	5.6	1	1.9	0.618
Abortion	10	18.5	10	18.5	0.999
IUGR	2	3.7	2	3.7	0.999

**Figure 1:**
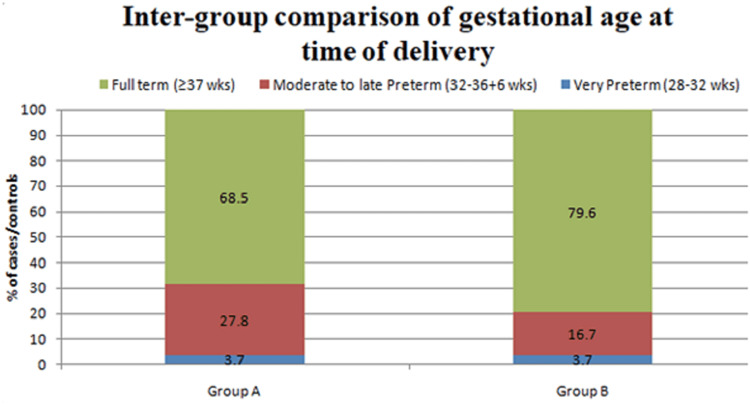
Inter-group comparison of gestational age at the time of delivery

Distribution of gestational ages at the time of delivery among the cases and control in study groups demonstrated no significant statistical difference with the distribution as shown in Figure 1.

The range of birth weights was considered 1000.0-2210.0 grams (gm) for Group A and 1020.0-3700.0 gm for Group B. The mean and standard deviation of birth weight of cases in Group A and Group B were 1906.7±327.7 and 2724.1±428.4 respectively stating a considerably higher mean birth weight distribution of Group B than Group A in the study (p=0.001) as shown in Table 3.

**Table 3:** Inter-group comparison of birth weight as a risk factor

	Group A (cases n=54)		Group B (controls n=54)		p-value
	Mean	Standard deviation	Mean	Standard deviation	
Birth weight (gram)	1906.7	327.7	2724.1	428.4	0.001

## Discussion

This case-control study focused on determining the risk factors associated with IUGR at a tertiary care hospital. In the present study, the majority of IUGR cases were found in women belonging to the younger age group (21 to 25 years) and Sinha and Kurude also reported similar results [9]. In a study by Romo et al., the average age of the mothers was 20.2+/-2.85 years, and young maternal age and < 20 years comprised 46.2%. Hence, young maternal age was identified as an independent risk factor for fetal growth restriction compared to middle-aged and older mothers [10]. 

According to research by Ashwani et al. [11], Sinha and Kurude [9], and Singh and Ambujam [12], a higher prevalence of IUGR has been associated with low socioeconomic status. Maternal health and nutrition are impacted by socioeconomic factors such as housing quality, employment, education level, and water supply source. In our study, 59.3% of cases belonged to the upper-lower class, and low socioeconomic status was found to be a significant factor affecting fetal growth. In the present study, there was even distribution of women with respect to parity status in the study group. According to Motghare et al., primiparity is positively correlated with an increased risk of IUGR [13]. A study by Ashwani et al. has shown multiparity as a significant factor in developing IUGR [11].

The majority of studies conducted on maternal risk factors of IUGR showed that anemia and hypertensive disorders of pregnancy were significant causes of fetal growth restriction supporting the findings of the current study. However, Ashwani et al. found that antepartum hemorrhage was one of the important risk factors of IUGR which was not significant in the current study [11]. 

In the studies conducted by Mohammad et al., a significant association was found between IUGR and previous history of abortion [14]. Many studies in literature like studies conducted by Shrestha et al. found higher recurrence in subsequent pregnancies for mothers who had IUGR infants in prior pregnancies [15]. In the current study, maternal obstetric and medical history like the history of abortion, IUGR, infection, and perinatal/neonatal mortality were found to be insignificant contributory factors for developing IUGR. Seravalli et al. also reported that the history of conception with assisted reproductive techniques was found more in the cases as compared to the controls but the statistical difference was insignificant [16]. The limitations of the study mainly involved a small sample size and a limited duration of the study which can be considered for the future scope of the present study.

## Conclusions

The most common obstetric problem, IUGR is frequently misdiagnosed antenatally. As a result, it is advised to use precise dates, offer early registration with routine prenatal checkups, clinical-sonographic examination, and correlation for fetal development in high-risk patients, and maintain strict antepartum surveillance when IUGR has been identified.

The study concludes that low socioeconomic status is a risk factor for IUGR owing to the poor living facility, lower literacy rate, and lack of awareness. The study also concludes that anemia and hypertensive disorders of pregnancy are the potential risk factors for IUGR on the grounds of nutritional deficiency and inadequate growth environment respectively. The maternal risk factors along with past medical and obstetric history may lead to IUGR. However, the reduced birth weight at the time of delivery should be taken into consideration for the risk factor of IUGR.
